# Vitamin D Deficiency in a Multiethnic Healthy Control Cohort and Altered Immune Response in Vitamin D Deficient European-American Healthy Controls

**DOI:** 10.1371/journal.pone.0094500

**Published:** 2014-04-11

**Authors:** Lauren L. Ritterhouse, Rufei Lu, Hemangi B. Shah, Julie M. Robertson, Dustin A. Fife, Holden T. Maecker, Hongwu Du, Charles G. Fathman, Eliza F. Chakravarty, R. Hal Scofield, Diane L. Kamen, Joel M. Guthridge, Judith A. James

**Affiliations:** 1 Arthritis and Clinical Immunology, Oklahoma Medical Research Foundation, Oklahoma City, Oklahoma, United States of America; 2 Departments of Medicine and Pathology, University of Oklahoma Health Sciences Center, Oklahoma City, Oklahoma, United States of America; 3 Division of Immunology and Rheumatology, Stanford University School of Medicine, Stanford, California, United States of America; 4 Division of Rheumatology, Medical University of South Carolina, Charleston, South Carolina, United States of America; University of Cape Town, South Africa

## Abstract

**Objective:**

In recent years, vitamin D has been shown to possess a wide range of immunomodulatory effects. Although there is extensive amount of research on vitamin D, we lack a comprehensive understanding of the prevalence of vitamin D deficiency or the mechanism by which vitamin D regulates the human immune system. This study examined the prevalence and correlates of vitamin D deficiency and the relationship between vitamin D and the immune system in healthy individuals.

**Methods:**

Healthy individuals (n = 774) comprised of European-Americans (EA, n = 470), African–Americans (AA, n = 125), and Native Americans (NA, n = 179) were screened for 25-hydroxyvitamin D [25(OH)D] levels by ELISA. To identify the most noticeable effects of vitamin D on the immune system, 20 EA individuals with severely deficient (<11.3 ng/mL) and sufficient (>24.8 ng/mL) vitamin D levels were matched and selected for further analysis. Serum cytokine level measurement, immune cell phenotyping, and phosphoflow cytometry were performed.

**Results:**

Vitamin D sufficiency was observed in 37.5% of the study cohort. By multivariate analysis, AA, NA, and females with a high body mass index (BMI, >30) demonstrate higher rates of vitamin D deficiency (p<0.05). Individuals with vitamin D deficiency had significantly higher levels of serum GM-CSF (p = 0.04), decreased circulating activated CD4^+^ (p = 0.04) and CD8^+^ T (p = 0.04) cell frequencies than individuals with sufficient vitamin D levels.

**Conclusion:**

A large portion of healthy individuals have vitamin D deficiency. These individuals have altered T and B cell responses, indicating that the absence of sufficient vitamin D levels could result in undesirable cellular and molecular alterations ultimately contributing to immune dysregulation.

## Introduction

The prevalence and significance of vitamin D deficiency has received significant attention in recent years. Reports of vitamin D deficiency prevalence vary depending on the population demographics [1]. Special emphasis has been placed on the prevalence of deficiency in populations thought to be at higher risk including individuals living at northern latitudes, the elderly, postmenopausal women receiving treatment for osteoporosis, and ethnic minorities, where incidences of 25-hydroxyvitamin D [25(OH)D]deficiency range from 30% to >50% [1 7]. Several factors contribute to the elevated risk of vitamin D deficiency including ethnicity, gender, age, residence in areas of low natural ultraviolet B irradiation (UVB), increased body mass index (BMI), and genetic variations in vitamin D metabolism pathways and vitamin D binding protein [5], [7], [8], [9], [10], [11], [12], [13], [14]. However, due to the wide spread variability of reported vitamin D deficiency, it is of interest to further examine potential risk factors for and the prevalence of vitamin D deficiency in a multiethnic cohort in the same location with a range of UVB seasonal variation, such as central Oklahoma at the 35^o^N latitude.

While the skeletal effects of vitamin D deficiency are well accepted, a growing body of research has begun to examine extraskeletal effects of vitamin D [1], [15], [16], [17], [18], [19], [20], [21], [22], [23], [24], [25], [26]. Vitamin D deficiency has been associated with cancer, cardiovascular disease, autoimmune diseases, type 2 diabetes, and infectious diseases particularly tuberculosis (TB) infection [27], [28], as well as all-cause mortality [1], [18], [19], [22], [23], [24], [25], [29], [30], [31], [32], [33], [34], [35]. Vitamin D receptors (VDRs) and vitamin D 1-α hydroxylase (CYP27B1), a necessary enzyme for vitamin D activation, is found in activated lymphocytes, macrophages, and dendritic cells and suggests an immunomodulatory role of vitamin D [26], [36], [37]. Previous *in vitro* and *ex vivo* studies demonstrate that vitamin D can effectively enhance innate anti-microbial responses and suppress adaptive immunity [38], [39], [40], [41].

Immunomodulatory roles of vitamin D can extend to the regulation of the proliferation and development of many immune cell subsets. Vitamin D modulates adaptive immune responses by inhibiting the T helper (Th)1 and Th17 cells [42], [43], [44], [45] and altering the activities of naïve B cells and antigen presenting cells (APCs) in both human and mouse [46], [47], [48]. Vitamin D has been demonstrated to skew the T cell populations toward increased numbers of regulatory T cells (Tregs) [49], [50], [51] and can enhance and maintain Treg induction [52], [53], [54], [55], [56]. Vitamin D is also paramount in the proper maturation of invariant natural killer T (iNKT) cells in mice that are capable of direct cytotoxic elimination of self-reactive cells [57]. Although the effects of vitamin D on B cell functions and differentiation have not been investigated extensively in human studies *in vitro*, vitamin D can suppress mature B cell proliferation, B cell cytokine secretion, plasma cell differentiation, and antibody production [43], [58], [59], [60], [61]. Early studies have demonstrated that active form of vitamin D (1α,25-dihydroxylcholecalciferol) can directly enhance the expansion of monocyte and myeloid cell line derived macrophages [62], [63], [64]. Vitamin D can also regulate the immune system via cytokine modulation. Several studies have shown that vitamin D can skew the cytokine profile from T helper (Th)1 and Th17 to Th2 [40] and enhance interleukin (IL)-10 secretion while reducing IL-2, IFN-γ, GM-CSF levels in activated lymphocytes *in vitro* [65], [66], [67] and in a vitamin D clinical trial [68]. Vitamin D deficiency is also associated with decreased frequency of IL-2-secreting CD8+ T cells [69], [70]. Additionally, seasonality-driven vitamin D3 variations in healthy adults correlate with changes in cytokine production and phenotype in the T cell compartment [71], [72].

The goal of this study was to examine the prevalence of vitamin D deficiency in a multiethnic cohort and to evaluate the impact of severe vitamin D deficiency on the healthy human immune system. This study provides potential insights for autoimmune and infectious disease etiology and identifies correlates of vitamin D deficiency.

## Materials and Methods

### Study subjects

Experiments were approved by the Institutional Review Board at the Oklahoma Medical Research Foundation and performed in accordance with the Helsinki Declaration. Healthy individuals (total participants, n = 774; European-American, EA, n = 470; Native American, NA, n = 179; African-American, AA, n = 125) were recruited over the course of one year at 15 health control fairs in central Oklahoma (35^o^N latitude). Participant demographics are summarized in **Table 1**. All study participants provided written informed consent prior to enrollment. Blood specimens were procured and medical record information including gender, age, self-reported ethnicity; height, weight, current medication use, and supplement use were collected. Serum was isolated and stored at -80°C until used. Peripheral blood mononuclear cells (PBMCs) were isolated using Lymphocyte Separation Medium (Mediatech, Inc, Manassas, VA) and stored in freezing media (20% human serum and 10% DMSO in RPMI) at −80°C until use. The UV index climatological mean (UVI) for the state of Oklahoma was calculated for each month of patient recruitment based on reports from the United States Environmental Protection Agency (EPA) (http://www.epa.gov/sunwise/uvimonth.html). UVB data for single wavelength at 305 nm at noon was acquired via the US National Aeronautics and Space Administration's (NASA's) Giovanni data set (http://disc.sci.gsfc.nasa.gov/giovanni, 2013 NASA Goddard Centre, Greenbelt, MD). Monthly average of UVB at 305 nm (slightly overestimated the prime physiological active wavelength of 290–295 nm) in Oklahoma was used for the further analysis. Analyses and visualization of UVB at 305 nm data used in this publication were produced with the Giovanni online data system, developed and maintained by NASA GES DISC.

**Table 1:** Demographics of all study participants.

	Total (n = 774)	European-American	Native American	African-American	p-value
		(n = 470)	(n = 179)	(n = 125)	
Male	246	164 (34.89%)	46 (25.70%)	36 (28.80%)	0.059
Female	528	306 (65.11%)	133 (74.30%)	89 (71.20%)	-
Age^b^, mean (range)	39.0 (18.0–86.0)	39.5 (19.0–86.0)	41.6 (19.0–74.0)	33.8 (18.0–62.0)	<0.001
BMI^a^ ^,b^, mean (range)	29.0 (16.5–58.4)	28.1 (16.5–58.4)	30.9 (18.3–56.7)	29.7 (17.4–51.6)	<0.001
Obese	236	150 (31.91%)	86 (48.04%)	53 (42.40%)	<0.001
Morbidly Obese	57	24 (5.11%)	20 (11.17%)	13 (10.40%)	0.011

a BMI = Body Mass Index; ^b^variables included in the multivariate regression analysis.

### 25(OH)D determination

Plasma 25(OH)D levels were determined in duplicate using a commercial enzyme immunoassay (Immunodiagnostic Systems, Inc., Scottsdale, AZ) according to manufacturer instructions, performed at the Oklahoma Center of Biomedical Research of Excellence (COBRE) Serum Analyte and Biomarker Core. Vitamin D sufficiency was defined as ≥20 ng/mL, deficiency as <20 ng/mL, and severely deficiency as <12 ng/mL [73]. In order to determine the effect of vitamin D on immune cell functions, we identified individuals with vitamin D levels above the 80^th^ percentile (>24.8 ng/ml) as ‘vitamin D sufficient’ and those with vitamin D levels below the 20^th^ percentile (<11.3 ng/ml) as ‘vitamin D severely deficient’.

### Cytokine assays

A 51-plex cytokine assay was used to measure CD40L, ENA78, eotaxin, bFGF, G-CSF, GM-CSF, gro alpha, hepatocyte growth factor, ICAM-1, IFN-α, IFN-β, IFN-γ, IL-10, IL-12p70, IL-12p40, IL-13, IL-15, IL-17, IL-17F, IL-1α, IL-1β, IL-1RA, IL-2, IL-4, IL-5, IL-6, IL-7, IL-8, IP10, leptin, LIF, M-CSF, MCP-1, MCP-3, MIG, MIP-1β, MIP-1α, nerve growth factor, PAI-1, PDGF-BB, RANTES, resistin, stem cell factor, soluble Fas ligand, TGF-α, TGF-β, TNF-α, TNF-β, TRAIL, VCAM-1, and VEGF in serum samples at the Stanford Human Immune Monitoring Center (http://iti.stanford.edu/research/himc-protocols-immunoassays.html).

Briefly, 96-well filter-bottom plates were wet with buffer and beads conjugated with capture antibodies for each cytokine were added. Sera samples, cytokine standards, and control sera were added in duplicate followed by incubation for two hours at room temperature and then for 18 hours at 4C°. Plates were washed and incubated with a biotin-labeled detector antibody for two hours. Next, the plates were washed and incubated with streptavidin-PE, followed by a final wash and re-suspension in reading buffer. Samples were read and data collected (100 beads per analyte) on the Luminex MAP200 instrument (Luminex Madison, WI). Data was analyzed using MasterPlex software (Hitachi Software Engineering America Ltd., MiraiBio Group, San Francisco, CA). Cytokines that fell below the detection limit of 1 pg/mL were assigned a near-zero value of 0.001 pg/mL for rank-based analyses. The cytokines that had more than 70% of the values below the detection limit (IFN-α, IL-10, IL1α, IL-6, and M-CSF) were excluded from the analysis. AssayChex beads (AssayChex, Radix Biosolutions, Georgetown, TX) were used to provide quality assurance for: addition of biotinylated detector antibodies, addition of streptavidin-PE, instrument performance, and non-specific background fluorescence. Both total bead count (>2000) and individual bead counts (>40) were verified. Samples that fell below this threshold were flagged for possible exclusion. Best-fit standard curves for each analyte were established by the MasterPlex software curve fit analysis, which included log-log transformation and weighting applied to the lower end of the curve. A plate passed quality control if the mean plate coefficient of variation (CV) <15%, and if not more than 20% of duplicates have CV>25%. Mean inter-assay CV of multiplexed bead-based assays for cytokine detection has been shown to be 10–14% in published studies [74], [75].

High sensitivity C-reactive protein (hs-CRP) was assessed using hsCRP protein enzyme immunoassay test kit (Biomerica, Irvine, CA) following the manufacturer's instructions. Relative serum concentration was calculated based on the assay-specific standard curve generated according to the manufacturer's instructions. The hs-CRP assay was performed at the Oklahoma COBRE Serum Analyte and Biomarker Core.

### Flow cytometry

PBMCs were thawed in media (>80% viability was obtained), washed twice, and re-suspended at 1×10^7^ viable cells/mL. Cells were added to the wells (50 µL cells per well) and stained for 45 minutes at room temperature with viability stain antibodies to surface markers for B cell (CD3, CD19, CD20, CD24, CD27, CD38 and IgD), T cell (CD3, CD4, CD8, CD25, CD38, CD45RA, CD45RO, CD127, CCR4, CCR7 and HLA-DR) NK, dendritic cell (DC) and monocyte (CD3+CD19+CD20, CD11c, CD14, CD16, CD56, CD123 and HLA-DR) subsets. A standard panel of surface markers developed at The Stanford Human Immune Monitoring Center was used (Stanford, CA http://iti.stanford.edu/research/himc-protocols-flowcytometry.html). Cells were then washed three times with FACS buffer (PBS supplemented with 2% FBS and 0.1% sodium azide), and re-suspended in 200 µL FACS buffer. Cells were collected (100,000 lymphocytes per sample) using DIVA 6.0 software on an LSRII flow cytometer (BD Biosciences). Data analysis was performed using FlowJo v9.3 (FlowJo, Ashland, OR) by gating on live cells based on forward versus side scatter profiles, then on singlets using forward scatter area versus height, followed by cell subset-specific gating.

### Cellular stimulation and phosphoepitope flow cytometry

PBMCs were thawed in media (>80% viability was obtained), washed twice and re-suspended at 0.5×10^6^ viable cells/mL. PBMCs (200 µL per well) were plated in 96-well deep-well plates. After resting for 1 hour at 37°C, cells were stimulated by 50 µL of cytokine (IFN-α, IFN-γ, IL-6, IL-7, IL-10, IL-2, or IL-21) with a final concentration of 50 ng/mL with an exception of IFN-α, which was 1×10^4^ units/mL, and incubated at 37°C for 15 minutes. The PBMCs were then fixed with paraformaldeyde, permeabilized with methanol, and kept at −80°C overnight. Each well was barcoded (stained with a unique combination of viability stain fluorescent dyes) using a combination of Pacific Orange and Alexa-750 (Invitrogen, Carlsbad, CA) and pooled in tubes. The cells were washed with FACS buffer and then stained with the following antibodies (all from BD Biosciences, San Jose, CA): CD3 Pacific Blue, CD4 PerCP-Cy5.5, CD20 PerCp-Cy5.5, CD33 PE-Cy7, CD45RA Qdot 605, phosphorylated STAT-1 (pSTAT-1) AlexaFluor488, pSTAT-3 AlexaFluor647, and pSTAT-5 PE. The samples were then washed and re-suspended in FACS buffer. Data from cells were collected (100,000 cells per stimulation condition) using DIVA 6.0 software on an LSRII flow cytometer (BD Biosciences). Data analysis was performed using FlowJo v9.3 by gating on live cells based on forward versus side scatter profiles, then on singlets using forward scatter area versus height, followed by cell subset-specific gating. Cell stimulation and phosphoepitope flow cytometry were performed at The Stanford Human Immune Monitoring Center (http://iti.stanford.edu/research/himc-protocols-flowcytometry.html).

### Statistical analyses

For normally distributed data, unpaired Student's t test was used. Pearson's correlations were used to analyze associations between cytokine and 25(OH)D levels. Cytokine, 25(OH)D levels, and a portion of phosphoflow cytometry results were non-normally distributed; therefore Mann Whitney and/or Kruskal-Wallis tests were performed when comparing multiple groups. Analyses were performed using GraphPad Prism 6.02 for Windows (GraphPad Software, San Diego, CA, USA). Multivariate logistic regression analyses were performed using SAS STAT 9.3 and R version 3.0.2 to predict the levels of serum vitamin D based on demographic and clinical information. In order to explicitly account for BMI, we conducted a nonparametric analysis of covariance, where each quantitative variable was transformed into ranks. Statistical significance was adjusted for multiple comparison testing using the False Discovery Rate method (FDR).

## Results

### Greater than half of individuals tested were vitamin D deficient and the majority of individuals of African-American ethnicity were vitamin D severely deficient

**Figure 1:**
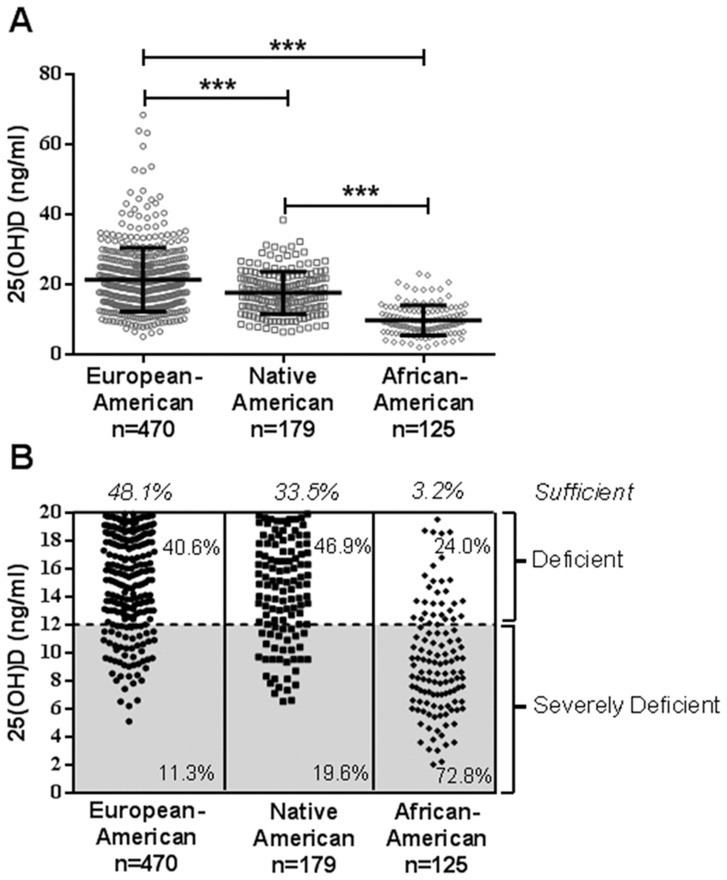
African-Americans and Native Americans have decreased 25(OH)D levels. (A) Median 25(OH)D levels for African-Americans (n = 125), Native Americans (n = 179), and European-Americans (n = 470). Error bars represent interquartile range. ***p<0.001, Kruskal-Wallis test with Dunn's multiple comparison; p<0.05, Shapiro-Wilk and D'Agostino normality test. (B) Frequency of vitamin D severe deficiency (<12 ng/mL) and vitamin D deficiency (12–20 ng/mL) in African-Americans, Native Americans, and European Americans. Each symbol represents one individual.

Plasma from 774 individuals was measured for 25(OH)D. Of the 774 individuals tested, 290 individuals (37.5%) were sufficient, 405 individuals (39.4%) were deficient, and 179 individuals (23.1%) were severely deficient. Median (inter quartile range [IQR]) 25(OH)D levels of 19.70 (15.20–25.33) ng/mL for EA, 17.20 (13.10–21.80) ng/mL for NA, and 9.20 (6.55–12.40) ng/mL for AA were observed (**Figure 1A**). Among the three ethnicities, African-Americans had the lowest level of 25(OH)D. Native Americans had significantly higher 25(OH)D levels than African-Americans (p<0.001) and 25(OH)D levels significantly lower than that of the European-Americans (p<0.001) (**Figure 1A**). The majority (62.5%) of the entire cohort had insufficient 25(OH)D levels and 72.8% of the African-Americans, 19.6% of the Native Americans, and 11.3% of the European-Americans were vitamin D deficient (**Figure 1B**).

### Male gender, increased BMI in females, UV index in Native Americans, and vitamin intake are associated with vitamin D levels

**Figure 2:**
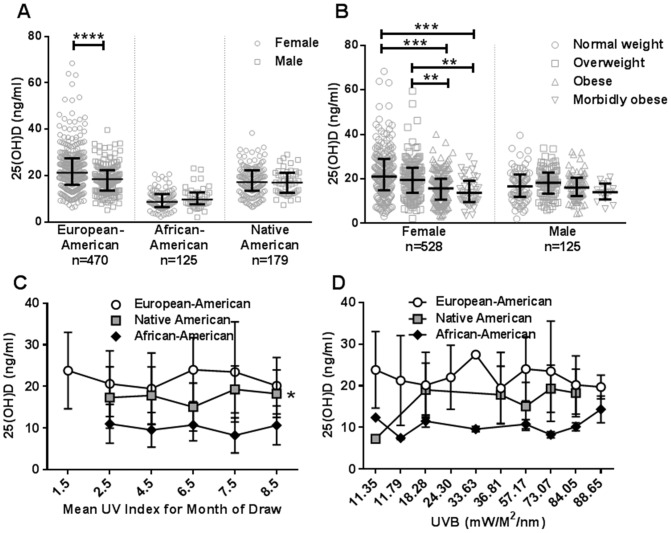
Male gender in European-Americans, increased BMI in females, and UV index in Native Americans are associated with vitamin D status. (A) Median 25(OH)D levels in males vs. females stratified by ancestral background. ****p<0.0001, Mann Whitney test; p<0.05, Shapiro-Wilk and D'Agostino normality test. (B) Median 25(OH)D levels in normal weight (BMI<25), overweight (BMI 25–30), obese (BMI 30–40), and morbidly obese (BMI>40) individuals stratified by gender. **p<0.01, ***p<0.001, Kruskal-Wallis test with Dunn's multiple comparison; p<0.05, Shapiro-Wilk and D'Agostino normality test. Error bars indicate interquartile range. (C) Mean 25(OH)D levels of individuals based upon the average UV index during the month in which their biological sample was obtained stratified by ethnicity. Error bars indicate SD; *r^2^ = 0.04, p<0.05. (D) Mean 25(OH)D levels of individuals based upon the average UVB 305 nm during the month in which their biological sample was obtained stratified by ethnicity. Error bars indicate SD.

Males had significantly lower levels of 25(OH)D compared to females (p = 0.013) and were found to be 1.65 times more likely to be vitamin D deficient as compared to females (p = 0.002, 95% confidence interval (CI) 1.20–2.29) (**Table 2**). However, when stratified by ethnicity, differences between 25(OH)D between genders were seen only in European-Americans (**Figure 2A**). European-American males (n = 164) had lower levels of vitamin D compared to females (n = 306) (males: median (IQR) 25(OH)D of 18.46 (13.50–22.35) ng/mL vs females: 21.23 (16.03–27.51) ng/mL (p<0.0001). This suggests that the overall 25(OH)D disparity between genders was primarily driven by European-Americans.

**Table 2:** Univariate analysis of study participants with 25(OH)D values <20 ng/mL and >20 ng/mL.

	25(OH)D			
	<20 ng/mL	>20 ng/mL	Odds Ratio	p-value*
	*n = 484*	*n = 290*	(95% CI^b^)	
Gender^d^				
Male	173 (70.00%)	73 (30.00%)	***1.65 (1.20–2.29)***	***0.002***
Female	311 (59.00%)	217 (41.00%)	***0.60 (0.44–0.84)***	***0.002***
Ethnicity^d^				
European-American	244 (52.00%)	226 (48.00%)	***0.29 (0.21–0.40)***	***<0.001***
Native American	119 (66.00%)	60 (34.00%)	1.25 (0.88–1.78)	0.219
African-American	121 (97.00%)	4 (3.00%)	***23.8 (8.70–65.3)***	***<0.001***
Vitamin use^d^	15 (33.00%)	31 (67.00%)	***0.27 (0.14–0.50)***	***<0.001***
Age^d^, median (range)	36 (18–84)	38 (19–86)		0.200
BMI^a,d^, median (range)	29 (17–58)	26 (17–50)		***<0.001***
Estrogen Use in females^d^	20 (32%)	43 (68%)	***0.28 (0.16–0.49)***	***<0.001***
UV index^c,†,d^, median (range)	5.50 (1.50–9.00)	4.50 (1.50–9.00)		***0.015***
UVB^d^ (mW/m^2^/nm)	46.99 (11.35–88.65)	36.81 (11.35–88.65)		***0.019***

*All categorical variables were analyzed by Fisher's exact test. Continuous variables were analyzed by Mann Whitney test. ^†^ Mean UV index during month of blood draw in Oklahoma, residence of all participants. ^a^BMI = Body Mass Index; ^b^CI = Confidence Interval; ^c^UV = Ultraviolet, Vitamin use refers to self-reported multivitamin use with vitamins containing vitamin D, or calcium with vitamin D; ^d^variables included in the multivariate regression analysis.

Vitamin D deficiency has previously been shown to be highly associated with BMI [14], [76], [77], [78], [79]. Here, we examined the association between BMI and vitamin D levels in a multiethnic cohort stratified by gender. Although 25(OH)D deficient individuals had higher BMI median than sufficient individuals in the cohort (p<0.001, **Table 2**), the difference in BMI between sufficient and deficient groups was largely accounted for in females (p<0.05) (**Figure 2B**). Further, the final multivariate regression model showed that there is a significant interaction between BMI and gender (p = 0.021, OR (CI) = 0.93 (0.88–0.99), **Table 3**).

**Table 3:** Multivariate logistic regression model.

Parameter	Odds Ratio (95% CI^b^)	p-value	β coefficient (SE^c^)
**25 (OH)D <20 ng/mL**			
Native American Ethnicity	1.52 (1.03–2.26)	0.036	0.42 (0.20)
African-American Ethnicity	26.10 (9.40–72.50)	<0.001	3.26 (0.52)
Male gender	12.60 (2.26–69.60)	0.004	2.53 (0.87)
BMI^a^	1.11 (1.07–1.14)	<0.001	0.10 (0.02)
Vitamin use	0.28 (0.14–0.56)	<0.001	−1.29 (0.36)
Estrogen use	0.50 (0.27–0.91)	0.025	−0.70 (0.31)
BMI*Male gender	0.93 (0.88–0.99)	0.021	−0.07 (0.03)

a BMI = Body Mass Index; ^b^CI = Confidence Interval; ^c^SE = Standard Error.

In an addition to gender and BMI, we also assessed the effect of average monthly UV index measured by EPA, 305 nm UVB exposure (**Figure S1 in File S1**), and vitamin supplement usage (an exogenous source of vitamin D). We found a weak positive correlation between UV index and 25(OH)D levels in Native Americans (r^2^ = 0.04, p<0.05) (**Figure 2C**). Similarly, we observed a trend toward a positive correlation between UVB and 25(OH)D levels (r^2^ = 0.02, p = 0.06) in Native Americans (**Figure 2D**). Both UV index and UVB irradiation at 305 nm were significantly different between vitamin D sufficient and insufficient groups (p = 0.015 and p = 0.019 respectively, **Table 2**).

**Figure 3:**
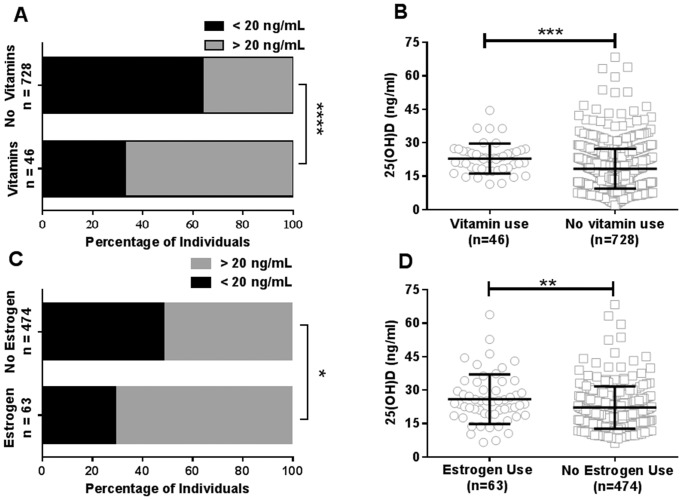
Vitamin usage and estrogen supplementation are associated with increased vitamin D levels. (A) Percentage of individuals that reported taking vitamins or not taking vitamins with 25(OH)D levels <20 ng/mL or >20 ng/mL. ****p<0.0001, Fisher's exact test. (B) Levels of 25(OH)D observed in individuals with self-reported vitamin use (n = 46) or no vitamin use (n = 728). Median 25(OH)D levels with interquartile range is shown. ***p<0.001, Mann Whitney test; p<0.05, Shapiro-Wilk and D'Agostino normality test. (C) Percentage of European-American female study participants that reported estrogen supplementation. *p<0.05, Fisher's exact test. (D) Levels of 25(OH)D in European-American female study participants with self-reported estrogen supplementation (n = 63) or no estrogen supplementation (n = 474). Median vitamin D levels with interquartile range are shown. **p<0.01, Mann Whitney test; p<0.05, Shapiro-Wilk and D'Agostino normality test.

Forty-six (5.9%) individuals reported taking supplements containing vitamin D (**Table 2**). Vitamin use was included if the self-reported medications contained multivitamins, vitamin D, or calcium with vitamin D and excluded if vitamin complexes did not contain vitamin D. Of the individuals reporting vitamin use, 67% had sufficient 25(OH)D levels [median (IQR):22.8 ng/mL (18.38–26.08)] compared to 33% of those not taking vitamins (median (IQR) 17.7 (12.23–22.88) p<0.001, OR (CI) = 0.27 (0.14–0.50), **Table 2** and **Figures 3A and 3B**). In our cohort, 63 women reported the use of either oral contraceptives or hormone replacement therapies containing estrogen, 87% of which were European-American. In this group, women who reported use of estrogen-containing therapies had significantly higher median (IQR) 25(OH)D levels (24.15 (19.05–30.07) ng/mL) than European-American females not taking estrogens [20.26 (15.74–26.87) ng/mL, p = 0.008, **Figures 3C and 3D**].

### Multivariate logistic regression analysis identified ethnicity, BMI, gender, vitamin intake, and estrogen use as significant predictors of deficient 25(OH)D levels

Candidate factors for multivariate models were identified based upon current knowledge of vitamin D risk factors and statistically significant univariate analysis findings identified herein: sex, ethnicity, age, BMI, average UV index during the month of blood draw, reported vitamin D supplementation intake, and the use of estrogen-containing therapies were all included as candidate correlates of vitamin D deficiency. African-American ethnicity [OR 26.1 (95%CI 9.4–72.5), p<0.001], BMI [OR 1.1 (1.1–1.1), p<0.001], male gender [OR 12.6 (2.3–69.6), p = 0.004], and Native American ethnicity [OR 1.52 (1.03–2.26), p = 0.036] were identified as significant independent predictors of vitamin D deficiency. Vitamin supplementation [OR 0.28 (0.14–0.56), p<0.001] was found to be protective against vitamin D deficiency (**Table 3**). The significant interaction term between BMI and male gender (p = 0.021, β = −0.07) suggests that males with high BMI were less likely to be vitamin D deficient compared to females with high BMI.

### Individuals with severe vitamin D deficiency had significantly higher GM-CSF serum levels than individuals with sufficient vitamin D

**Figure 4:**
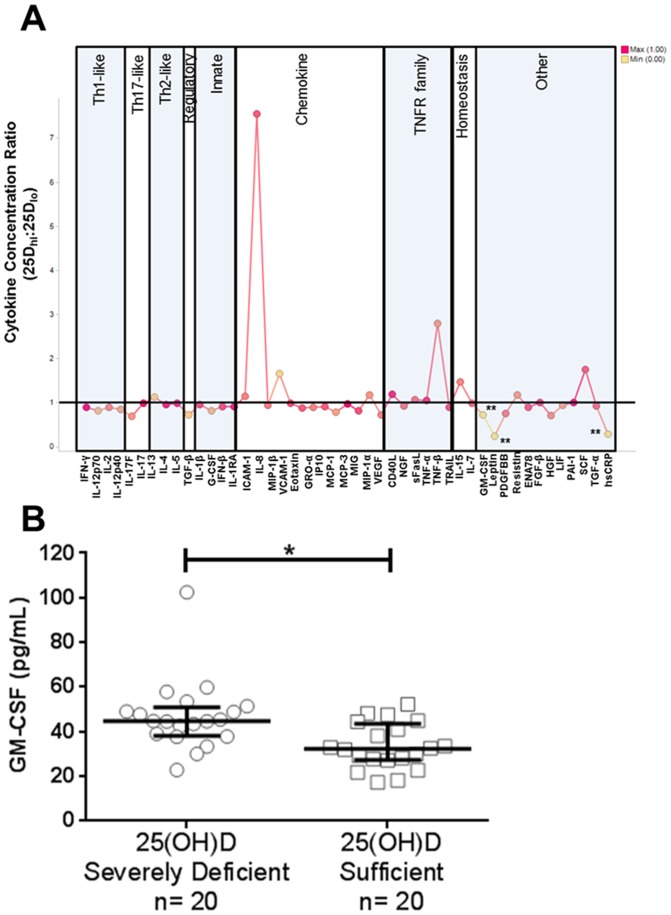
GM-CSF concentration was reduced in the vitamin D sufficient group. (A) Cytokine concentration ratio of vitamin D sufficient group to vitamin D severely deficient group. **p<0.01, Mann Whitney U Test. (B) Median concentration of GM-CSF in vitamin D severely deficient (n = 20) and sufficient groups (n = 20). *p<0.05, Mann-Whitney U Test corrected for BMI; p<0.05, Shapiro-Wilk and D'Agostino normality test. Error bars indicate interquartile range.

Previously published studies support the ability of vitamin D to modify the cytokine secretion profile of lymphoid and myeloid cells under various inflammatory states [16], [80]. To further characterize the effects of vitamin D on circulating soluble mediators including cytokines, chemokines, growth factors, and soluble receptors, we measured the concentrations of 52 serum cytokines in 20 healthy individuals whose vitamin D levels were below the 20^th^ percentile (<11.3 ng/mL, severely deficient) and 20 healthy individuals within the 80^th^ percentile (>24.8 ng/mL, sufficient). To control for the potential confounding variables, these 40 individuals were matched based on age, gender, and self-reported ethnicity (EA). Only EA study participants were further examined as the NA and AA study participants did not have enough individuals with sufficient vitamin D levels to allow for statistically significant observations. Demographics for these individuals are described in **Table 4**. Initial analysis showed that leptin, hsCRP, and GM-CSF were statistically higher (p-value<0.05) in vitamin D severely deficient individuals compared to vitamin D sufficient individuals (**Figure 4A** **and Table S1 in File S1**). However, both leptin and hsCRP have been shown to be associated with obesity [81], [82]. To ensure the observed associations were independent of adiposity, BMI was used in a conditional logistic regression analysis. This analysis showed that the differences in leptin and hsCRP between the two groups were confounded by BMI, whereas the difference in GM-CSF was BMI independent (**Table S1 in File S1**). GM-CSF concentrations were significantly higher in vitamin D severely deficient individuals as compared to vitamin D sufficient individuals (p = 0.04, **Figure 4B**). This observation is consistent with the results from stimulation assays *in vitro*, where the presence of vitamin D drastically reduced GM-CSF expression in peripheral blood lymphocytes [83].

**Table 4:** Demographics of the European-American individuals selected for cellular and biomarker assays.

Parameter	Severely Deficient Vitamin D	Sufficient Vitamin D	p-value
	(n = 20)	(n = 20)	
Vitamin D, median (range)	8.95 (6.15–10.36)	40.95 (30.97–63.91)	<0.01
Gender			
Male, count (%)	5 (25.00%)	5 (25.00%)	1.00
Female, count (%)	15 (75.00%)	15 (75.00%)	-
Age, median (range)	43.00 (24.00–62.00)	26.50 (21.00–69.00)	0.07
UV Index†, median (range)	4.50 (1.50–8.00)	4.50 (1.50–8.00)	0.88
UVB, median (range)	36.81 (11.35–84.05)	36.81 (11.35–84.05)	0.90
BMI^a^, median (range)	28.92 (20.91–43.26)	23.50 (17.16–31.00)	<0.01

† Mean UV index during month of blood draw in Oklahoma, residence of all participants. ^a^BMI = Body Mass Index.

### Severely vitamin D deficient individuals have decreased frequency of activated CD4+ and CD8^+^ T cells

**Figure 5:**
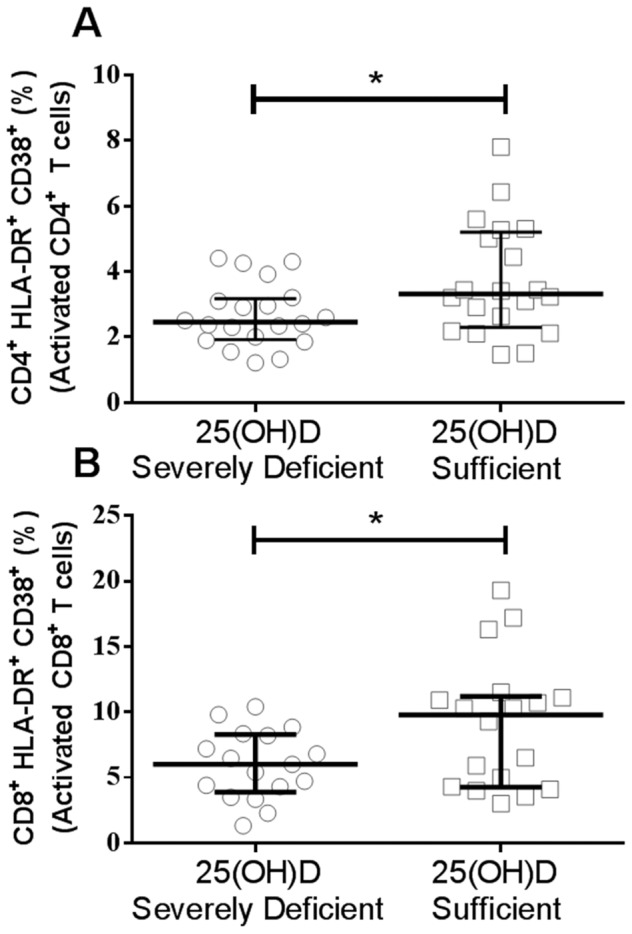
Activated CD4^+^ and CD8^+^ T cell levels are increased in the vitamin D sufficient group. (A) Percentage of activated CD4^+^ T cells (CD4^+^CD38^+^) in both vitamin D sufficient (n = 20) and severely deficient (n = 20) groups are shown. Mann Whitney U Test. (B) Percentage of activated CD8^+^ T cells (CD8^+^CD38^+^) in both vitamin D sufficient (n = 17) and severely deficient (n = 18) groups are shown. *p<0.05, Mann Whitney U Test; p<0.05, Shapiro-Wilk and D'Agostino normality test. Median and interquartile range are shown.

The presence of VDR has been observed on a variety of immune cell subtypes indicating vitamin D plays a role in immune cell signaling leading to their differentiation, activation, or maintenance [49]. To fully delineate the effects of vitamin D on immune cell frequencies and activation status in healthy individuals, peripheral blood mononuclear cells (PBMCs) from 40 individuals (20 vitamin D sufficient and 20 vitamin D severely deficient, **Table 4**) were immunophenotyped to assess the expressions of surface markers on PMBCs. Vitamin D sufficient individuals had significantly higher frequencies of activated (CD38^+^/HLA-DR+) CD4^+^ T cells as compared to the severely deficient vitamin D individuals at basal level (p = 0.04), as well as significantly higher frequencies of activated (CD38^+^/HLA^−^DR^+^) CD8^+^ T (p = 0.04) cells (**Figure 5** **A and B**, **Table S2 in File S1**). No significant differences in frequencies of B cell subsets and monocytes were observed between the two groups (**Table S2 in File S1**). These results indicate that vitamin D levels may affect T cell subset development and the activation capacity of cytotoxic and helper T cells *in vivo*.

### Vitamin D severely deficient individuals demonstrate decreased phosphorylated STAT1 in response to IL-2, IL-10, stimulation, but increased phosphorylated STAT1 in response to IFN-γ stimulation in CD4^+^ T cells

In order to elucidate the effects of vitamin D on CD4^+^ T and CD8^+^ T cell responses, phosphoepitope flow cytometry assays were performed to assess STAT1, STAT3, and STAT5 phosphorylation (pSTAT) status in the presence of both pro-inflammatory (IFN-α, IFN-γ, IL-6, IL-7, IL-2, and IL-21) and anti-inflammatory (IL-10) cytokines.

**Figure 6:**
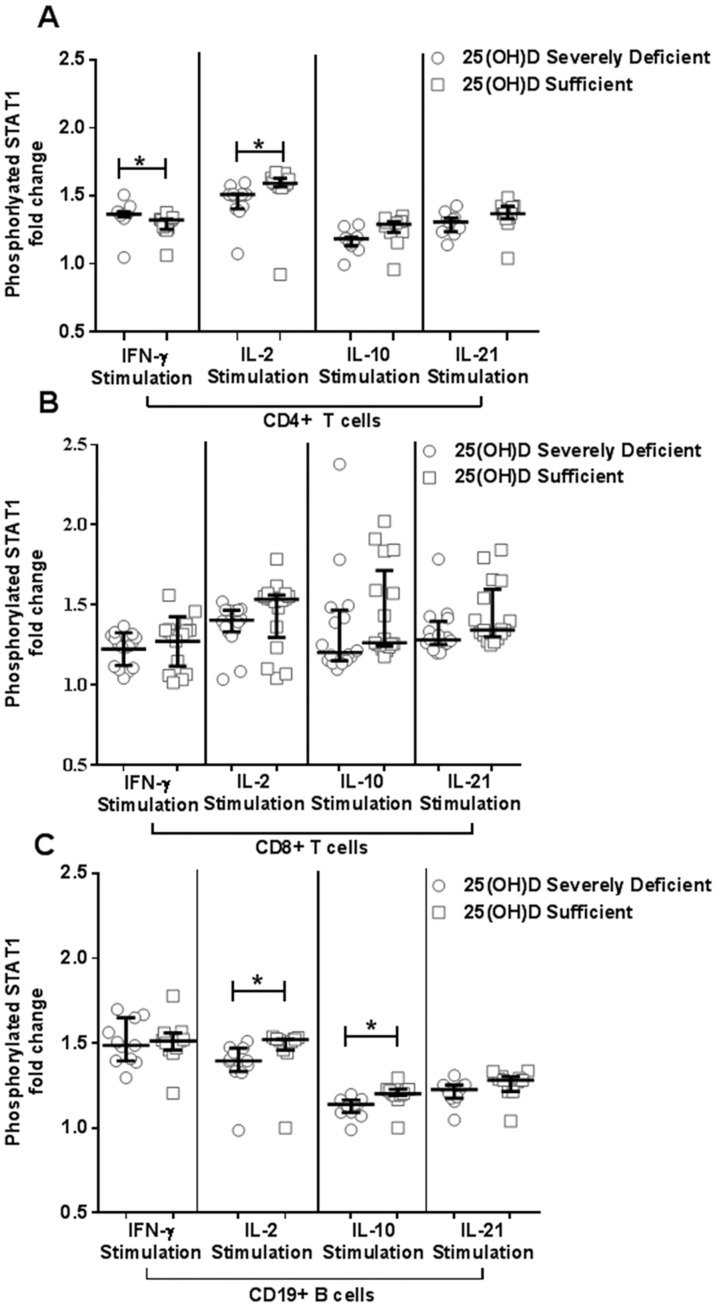
Phosphorylated STAT1 responses to IL-2 was increased in both CD4^+^ T cells and B cells from vitamin D sufficient group. (A) Median STAT1 phosphorylation (pSTAT1) fold changes after IFN-γ, IL-2, IL-10, and IL-21 stimulation in CD4^+^ T cells from both vitamin D sufficient (n = 11) and severely deficient (n = 11) groups. (B) Median STAT1 phosphorylation fold changes after IFN-γ, IL-2, IL-10, and IL-21 stimulation in CD8^+^ T cells from both vitamin D sufficient (n = 17) and deficient (n = 16) groups. (C) Median STAT1 phosphorylation fold changes after IFN-γ, IL-2, IL-10, and IL-21 stimulation in CD19^+^ B cells from both vitamin D sufficient (n = 11) and severely deficient (n = 11) groups. Mann Whitney U Test. *q<0.05 after BMI and multiple testing correction; p<0.05, Shapiro-Wilk and D'Agostino normality test. Error bars indicate interquartile range.

Individuals with severely deficient vitamin D levels as compared to individuals with sufficient vitamin D levels had an increased fold change of pSTAT1 (**Figure 6A**) in their CD4^+^ T cells in response to IFN-γ stimulation (p<0.01, q = 0.01) (**Table S3 in File S1**). Stimulation with IL-2 resulted in slightly increased fold change in pSTAT1 in CD4^+^ T cells of vitamin D sufficient individuals (p<0.01, q = 0.03, **Figure 6A**). CD8^+^ T cells from vitamin D severely deficient individuals trended toward decreased pSTAT1 in response to IL-2, IL-10, and IL-21 (p = 0.02 and q = 0.17, **Table S3** **in File S1** and **Figure 6B**). These results suggest that vitamin D can hinder the Th1 directed responses while enhancing the cellular responses to modulatory cytokines such as IL-2, IL-10.

### B cells from vitamin D severely deficient individuals demonstrated decreased levels of phosphorylated STAT1 in response to IL-2 and IL-10 stimulation

Several studies have implicated some suppressive effects of vitamin D on activated B cells either directly or indirectly through other immune cells [59], [84], [85]. We assessed differences in the fold-change in phosphorylation of STATs in B cells upon various cytokine stimulations in vitamin D severely deficient and vitamin D sufficient individuals. B cells from vitamin D sufficient individuals had increased pSTAT1 than vitamin D severely deficient individuals in response to IL-2 and IL-10 stimulation independent of BMI (p<0.001 and q<0.05, **Figure 6C**). These results suggest that vitamin D can modulate B cell responses to both anti- and pro-inflammatory stimuli.

## Discussion

In this study we detected a large proportion of healthy individuals to be vitamin D deficient (62.5%) in a multiethnic cohort containing African-Americans, European-Americans, and Native Americans. While many studies have demonstrated decreased 25(OH)D levels in African-Americans [9], [10], [86], this is the first report to describe the high level of vitamin D deficiency in Native Americans of Oklahoma.

Nearly two-thirds of the Native Americans in this study were deficient and approximately one-third of those individuals had severe vitamin D deficiency. Native American populations have a six-fold increase in mortality from tuberculosis and a three-fold increase in mortality from type 2 diabetes [87], both of which have been associated with vitamin D deficiency [1], [23], [88]. Therefore, addressing this deficiency in Native American populations is critical.

Contrary to previous reports of vitamin D deficiency in women [9], [89], European-American men were found to have significantly lower vitamin D levels as compared to European-American women. This could be in part due to the use of estrogens in women that was independently associated with sufficient 25-hydroxyvitamin D levels. Several studies have shown an association between oral contraceptive use and increased 25(OH)D levels [5], [90]. Further, we confirmed several reports demonstrating an association with obesity and decreased circulating 25(OH)D levels [5], [8], [9]. This association was seen only among females, possibly resulting from the differences in adipose tissue distribution in males and females.

Our study identified that 62.5% of study participants had vitamin D deficient and 23.2% of individuals were severely vitamin D deficient. The high prevalence of vitamin D deficiency found in this study, combined with reported low rates of vitamin D supplementation, emphasize the importance of increased awareness and supplementation, especially in populations at increased risk including men, obese individuals, African-Americans, and Native Americans.

Vitamin D has recently been recognized as a potent immune regulator [16], [26], [37], [40], [49] and is linked with the enhanced risk of developing several systemic autoimmune conditions, such as multiple sclerosis, rheumatoid arthritis, and systemic lupus erythematosus [91], [92]. Although the immediate effects of vitamin D on the immune responses have been dissected *in vitro*, the persistent cellular and molecular alterations *in vivo* during deficient vitamin D states in healthy humans are just beginning to be systematically addressed [71], [72]. Enhanced induction of cytotoxic T cells, Treg, Th2, and monocyte-derived macrophages by vitamin D has been well documented in literature [40], [49], [58], [92]. Flow cytometry analysis of the frequencies of different T and B cell subsets and monocytes in both vitamin D severely deficient and sufficient groups demonstrated an expansion of activated CD4^+^ and CD8^+^ T cells in the vitamin D high group (**Figure 5**). This finding is consistent with previous studies indicating an association between vitamin D and T cell activation [93], [94].

Vitamin D has been shown to modulate immune responses by altering T cells [42], [43], [44], [45], [49], [50], [51], naïve B cells, and APCs [46], [47], [48]. As such, we assessed leukocyte responses to various immunological stimuli in both vitamin D sufficient and severely deficient groups. Our results indicated that CD4^+^ T cells increased phosphorylation of STAT1 in response to IFN-γ, IL-2, and IL-10 (**Figure 6**). This observation was particularly intriguing, since both a strong interferon signature and vitamin D deficiency have been observed in individuals with autoimmune disease [29], [30], [31], [32], [33], [34], [35], [95], [96], [97], [98]. The modestly increased T cell responses to IL-2 and IL-10 along with slightly reduced response to IFN-γ in CD4^+^ T helper populations from the vitamin D sufficient group suggest that the presence of vitamin D may play a role in the attenuation of immunomodulatory responses.

The frequency of B cells was not significantly different between vitamin D sufficient and severely deficient groups (**Table S1 in File S1**). B cells from vitamin D high individuals had slightly higher responses to IL-2 and IL-10 (**Figure 6C**). IL-10 is critical for the induction and maintenance of regulatory B cells that are capable of secreting regulatory cytokines and inducing Treg differentiation [99], [100]. The modestly increased phosphorylation of STAT1 resulting from IL-10 stimulation in B cells from vitamin D sufficient individuals suggests that vitamin D may potentiate regulatory B cell induction, expansion, and maintenance. Following phosphorylation, STAT molecules can dimerize and translocate into the nucleus allowing for fine-tuning of biological responses [101]; thus, even modest changes in phosphorylation levels can lead to alterations in down-stream events. As this is a nested case pilot study, further characterization of the differences in immune responses between vitamin D sufficient and deficient individuals is warranted.

In this study, we found that a large portion of healthy individuals recruited in central Oklahoma (between 35^o^N and 37^o^N latitude) have vitamin D deficiency, particularly in European-American men and in individuals of African-American or Native American descent. To evaluate and explore biological pathways that might be affected by extremely low levels of vitamin D, we initially designed the immune response experiments for all three ethnicities. However, the high prevalence of vitamin D deficiency in both African-American and Native American individuals rendered a matched study design within these demographics impossible. As such, 20 vitamin D sufficient and 20 severely vitamin D deficient European-American individuals were selected for further study. As this is a pilot study, future studies to further characterize and examine mechanisms behind deficient vitamin D levels and potentially altered associated immune responses in a larger study cohort is warranted. Despite the limitations, our results show that low levels of vitamin D can associated with changes T and B cell responses, indicating that the sustained absence of sufficient vitamin D levels could contribute to immune dysregulations. Considering the multitude of ongoing clinical studies and trials on vitamin D supplementation in autoimmune inflammatory and other chronic disease, our findings may assist the community in testing immune-based, mechanistic hypotheses in these prospective studies.

## Supporting Information

File S1 **Figure S1 and Tables S1–S3.** Figure S1. UVB 305 nm levels in Oklahoma. UVB index of both northern and southern extreme areas in OK state was obtained via NASA UVB data (A). Differences in average monthly average UVB index between these two regions were displayed (B). Error bars indicate SEM. Table S1. Mann-Whitney analysis of cytokine concentration between vitamin D high and low groups. Table S2. Mann-Whitney rank-based analysis of immunophenotyping factors between vitamin D sufficient and deficient individuals. Table S3. Mann-Whitney rank-based analysis of phosphorylation of signaling transducers after various stimulation of immune cells in vitamin D sufficient and deficient individuals.
(DOC)
