# Supplementary material for: Vitamin D Deficiency in a Multiethnic Healthy Control Cohort and Altered Immune Response in Vitamin D Deficient European-American Healthy Controls
Source: PLoS One. 2014 Apr 11;9(4):e94500. doi: 10.1371/journal.pone.0094500 (PMC3984168; doi:10.1371/journal.pone.0094500)
Supplement: File S1 — Figure S1 and Tables S1–S3. Figure S1. UVB 305 nm levels in Oklahoma. UVB index of both northern and southern extreme areas in OK state was obtained via NASA UVB data (A). Differences in average monthly average UVB index between these two regions were displayed (B). Error bars indicate SEM. Table S1. Mann-Whitney analysis of cytokine concentration between vitamin D high and low groups. Table S2. Mann-Whitney rank-based analysis of immunophenotyping factors between vitamin D sufficient and deficient individuals. Table S3. Mann-Whitney rank-based analysis of phosphorylation of signaling transducers after various stimulation of immune cells in vitamin D sufficient and deficient individuals. (DOC) [file pone.0094500.s001.doc]

**Figure S1**

**
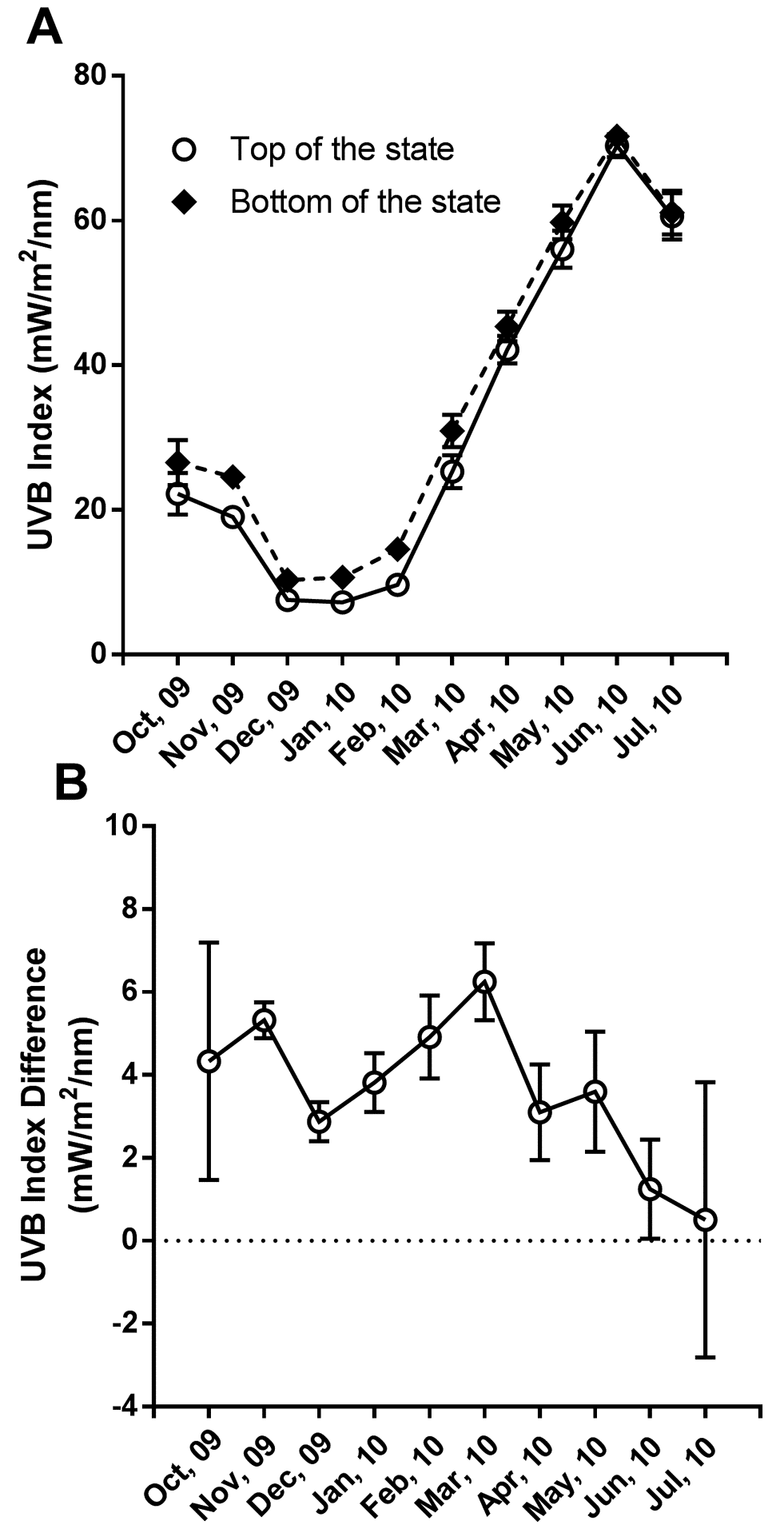
**

**Table S1. Mann-Whitney analysis of cytokine concentration between vitamin D high and low groups.**

| **Cytokine** | **Vitamin D Severely Deficient**  **Median (IQR)** | **Vitamin D Sufficient**  **Median (IQR)** | **p-value** | **Adjusted p-value^a^** |
| --- | --- | --- | --- | --- |
| **hsCRP** | 3016.84 (1274.81 - 6600.70) | 851.08 (399.96 - 1383.50) | **0.01** | 0.42 |
| **BLyS** | 919.81 (659.75 - 1015.35) | 899.40 (720.84 - 1136.21) | 0.84 | 0.43 |
| **CD40 Ligand** | 97.51 (68.01 - 135.22) | 115.72 (49.29 - 167.03) | 0.90 | 0.34 |
| **ENA78** | 122.2 (62.29 - 166.59) | 109.76 (57.31 - 173.80) | 0.69 | 0.64 |
| **Eotaxin** | 30.26 (21.82 - 41.65) | 30.00 (17.65 - 41.01) | 0.74 | 0.41 |
| **FGF-β** | 0.65 (0.22 - 3.32) | 0.65 (0.65 - 4.15) | 0.80 | 0.75 |
| **G-CSF** | 4.75 (0.86 - 7.47) | 3.88 (0.35 - 9.80) | 0.16 | 0.39 |
| **GM-CSF** | 44.72 (38.72 - 49.65) | 32.29 (27.63 - 41.87) | ***<0.01*** | ***0.04*** |
| **GRO ALPHA** | 10.42 (9.12 - 13.14) | 9.23 (8.23 - 19.06) | 0.65 | 0.71 |
| **HGF** | 50.14 (29.92 - 58.72) | 35.56 (21.46 - 52.51) | 0.39 | 0.86 |
| **ICAM-1** | 5156.38 (3622.29 - 8458.44) | 5890.42 (4917.39 - 7759.04) | 0.44 | 0.68 |
| **IFN-β** | 7.03 (2.64 - 21.57) | 6.39 (0.15 - 12.50) | 0.76 | 0.93 |
| **IFN-γ** | 13.96 (9.07 - 18.06) | 12.46 (7.95 - 20.74) | 0.99 | 0.51 |
| **IL-12-P70** | 5.86 (4.72 - 8.29) | 4.82 (4.11 - 5.60) | 0.15 | 0.43 |
| **IL-12P40** | 12.73 (10.60 - 14.85) | 10.86 (9.41 - 12.79) | 0.26 | 0.53 |
| **IL-13** | 6.735 (4.00 - 8.79) | 7.57 (6.63 - 10.04) | 0.07 | 0.11 |
| **IL-15** | 7.7 (2.50 - 23.48) | 11.38 (3.36 - 14.50) | 0.43 | 0.22 |
| **IL-17** | 4.44 (3.71 - 8.06) | 4.39 (3.89 - 5.39) | 0.95 | 0.91 |
| **IL-17f** | 11.65 (6.76 - 14.55) | 8.15 (3.02 - 14.36) | 0.34 | 0.37 |
| **IL-1β** | 1.89 (1.16 - 2.64) | 1.81 (1.23 - 3.12) | 0.78 | 0.63 |
| **IL-1RA** | 32.7 (1.22 - 78.22) | 30.07 (5.03 - 73.20) | 0.97 | 0.96 |
| **IL-2** | 74.93 (48.33 - 100.93) | 67.69 (45.54 - 79.42) | 0.45 | 0.31 |
| **IL-4** | 5.075 (3.59 - 6.66) | 4.89 (3.99 - 6.09) | 0.95 | 0.88 |
| **IL-5** | 36.87 (29.86 - 43.03) | 36.30 (29.70 - 45.71) | 0.92 | 0.7 |
| **IL-7** | 85.35 (72.18 - 120.02) | 84.08 (74.85 - 101.40) | 0.56 | 0.32 |
| **IL-8** | 4.11 (1.51 - 68.42) | 31.04 (1.28 - 139.11) | 0.65 | 0.68 |
| **IP10** | 27.52 (21.51 - 37.93) | 24.62 (19.71 - 32.53) | 0.44 | 0.64 |
| **LEPTIN** | 5411.43 (3025.03 - 9824.33) | 2225.49 (1322.74 - 3283.56) | ***<0.01*** | 0.05 |
| **LIF** | 9.48 (7.53 - 12.03) | 9.02 (7.62 - 10.25) | 0.27 | 0.54 |
| **MCP-1** | 29.49 (22.91 - 35.53) | 26.68 (19.49 - 39.35) | 0.65 | 0.42 |
| **MCP-3** | 24.97 (18.04 - 27.72) | 19.84 (17.23 - 27.76) | 0.26 | 0.33 |
| **MIG** | 15.54 (10.62 - 22.49) | 15.11 (9.48 - 26.95) | 0.98 | 1 |
| **MIP-1β** | 61.82 (36.59 - 99.72) | 58.10 (42.52 - 137.12) | 0.49 | 0.86 |
| **MIP-1α** | 20.49 (14.28 - 31.05) | 16.93 (12.88 - 35.28) | 0.71 | 0.95 |
| **NGF** | 45.43 (33.09 - 55.18) | 41.99 (33.08 - 49.15) | 0.53 | 0.52 |
| **PAI-1** | 1966.58 (1747.50 - 2217.28) | 1992.15 (1678.40 - 2517.06) | 0.99 | 0.71 |
| **PDGFBB** | 484.17 (266.13 - 666.02) | 368.84 (261.60 - 549.19) | 0.49 | 0.41 |
| **RANTES** | 298.82 (258.66 - 427.80) | 352.14 (233.13 - 498.69) | 0.35 | 0.55 |
| **Resistin** | 1340.45 (831.47 - 1543.58) | 1583.55 (789.81 - 1960.88) | 0.33 | 0.36 |
| **SCF** | 1.45 (0.43 - 3.86) | 2.56 (0.16 - 4.22) | 0.68 | 0.29 |
| **sFAS ligand** | 7.32 (6.06 - 8.90) | 7.77 (6.31 - 11.19) | 0.56 | 0.73 |
| **TGF-α** | 0.73 (0.35 - 1.55) | 0.68 (0.16 - 1.88) | 0.61 | 0.49 |
| **TGF-β** | 9.75 (7.17 - 12.12) | 7.13 (5.04 - 9.30) | 0.09 | 0.36 |
| **TNF-α** | 2.15 (1.48 - 3.22) | 2.26 (1.81 - 2.87) | 0.87 | 0.75 |
| **TNF-β** | 0.05 (0.00 - 0.27) | 0.14 (0.00 - 0.53) | 0.33 | 0.65 |
| **Trail** | 31.22 (24.17 - 37.49) | 28.20 (22.74 - 39.18) | 0.77 | 0.59 |
| **VCAM-1** | 582.65 (484.55 - 790.56) | 967.99 (665.03 - 1312.76) | 0.06 | 0.08 |
| **VEGF** | 30.13 (18.33 - 41.77) | 21.90 (17.56 - 35.52) | 0.46 | 0.54 |

^a^p-values are corrected for BMI using conditional logistic.

**Table S2. Mann-Whitney rank-based analysis of immunophenotyping factors between vitamin D sufficient and deficient individuals.**

| **Immunophenotyping** | **Vitamin D Severely Deficient Median (IQR)** | **Vitamin D Sufficient Median (IQR)** | **p-value^a^** | **q-value^b^** |
| --- | --- | --- | --- | --- |
| % monocytes | 13.05 (9.51 - 15.5) | 15.72 (14.55 - 21.69) | 0.18 | 0.96 |
| CXCR3^+^, % of monocytes | 10 (5.95 - 25.4) | 10.57 (4.97 - 34.83) | 0.54 | 0.96 |
| % lymphocytes | 85.5 (82.81 - 88.52) | 81.8 (77.69 - 84.4) | 0.22 | 0.96 |
| Lymphocyte:Monocyte Ratio | 6.55 (5.44 - 9.23) | 5.26 (3.5 - 5.69) | 0.16 | 0.96 |
| B cells, % of lymphocytes | 19.4 (12.35 - 36.73) | 20.65 (11.12 - 24.88) | 0.58 | 0.96 |
| Naïve B, % of B cells | 63.45 (57.3 - 80.92) | 70.05 (56.55 - 73.18) | 0.16 | 0.96 |
| Transitional B, % of B cells | 2.38 (1.84 - 3.61) | 2.45 (1.64 - 3.29) | 0.65 | 0.96 |
| Memory B, % of B cells | 15.55 (6.95 - 30.27) | 19.2 (13.72 - 25.43) | 0.42 | 0.96 |
| IgDPos/CD27^-^, % of B cells | 63.6 (45.18 - 72.9) | 57 (51.28 - 62.15) | 0.94 | 0.96 |
| IgDPos/CD27^+^, % of B cells | 8.2 (6.51 - 14.03) | 11.95 (10.3 - 16.88) | 0.43 | 0.96 |
| IgDNeg/CD27^+^, % of B cells | 13.95 (10.83 - 18.38) | 16.75 (14.72 - 20.12) | 0.84 | 0.96 |
| IgDNeg/CD27^-^, % of B cells | 11.75 (5.76 - 16.12) | 12.25 (9.4 - 15.42) | 0.92 | 0.96 |
| CXCR3^+^, % of B cells | 4.18 (1.71 - 5.4) | 3.14 (2.08 - 11.47) | 0.66 | 0.96 |
| Plasmablasts, % of CD3^-^/CD20^-^ lymphocytes | 3.85 (2.87 - 5.74) | 4.61 (2.03 - 6.72) | 0.99 | 0.99 |
| NK cells, % of lymphocytes | 19.55 (11.83 - 32.53) | 20.8 (12.05 - 26.97) | 0.56 | 0.96 |
| CD94^+^, % of NK cells | 51.23 (32.49 - 60.55) | 53.87 (38.26 - 61.59) | 0.57 | 0.96 |
| CD314^+^, % of NK cells | 87.9 (80 - 95.08) | 86.28 (82.9 - 96.45) | 0.29 | 0.96 |
| HLA-DR^+^, % of NK cells | 3.42 (0.7 - 6.65) | 7.23 (0.7 - 12.87) | 0.89 | 0.96 |
| CXCR3^+^, % of NK cells | 13.3 (7.36 - 17.35) | 14.92 (9.13 - 19.63) | 0.74 | 0.96 |
| NKT cells, % of lymphocytes | 5.68 (3.81 - 8.6) | 4.17 (3 - 5.86) | 0.59 | 0.96 |
| gamma-delta T cells, % of lymphocytes | 4.97 (2.52 - 8.82) | 7.87 (2.79 - 11.85) | 0.4 | 0.96 |
| T cells, % of lymphocytes | 73.95 (65.75 - 76.7) | 69.95 (66.7 - 72.08) | 0.77 | 0.96 |
| CD4^+^, % of T cells | 61.55 (57.97 - 71.82) | 63.45 (59.52 - 71.35) | 0.8 | 0.96 |
| CD27^+^/CD45RA^+^, % of CD4 T cells | 43.65 (30.23 - 51.53) | 47.55 (35.45 - 58.65) | 0.31 | 0.96 |
| CD27^+^/CD45RA^-^, % of CD4 T cells | 46.9 (39.6 - 55.83) | 38.4 (31.4 - 46.77) | ***0.04*** | 0.7 |
| CD27^-^/CD45RA^-^, % of CD4 T cells | 8.28 (4.25 - 11.5) | 8.25 (5.45 - 13.28) | 0.79 | 0.96 |
| CD27^-^/CD45RA^+^, % of CD4 T cells | 1.1 (0.48 - 3.41) | 1.64 (0.68 - 4.58) | 0.37 | 0.96 |
| CD28^+^, % of CD4 T cells | 98.8 (94.6 - 99.4) | 98.45 (96.52 - 99.23) | 0.53 | 0.96 |
| CD85^+^, % of CD4 T cells | 0.9 (0.27 - 1.8) | 1.2 (0.68 - 2.28) | 0.11 | 0.96 |
| PD-1^+^, % of CD4 T cells | 14.85 (6.59 - 22.2) | 14.4 (6.8 - 21.08) | 0.85 | 0.96 |
| HLA-DR^+^/CD38^+^, % of CD4 T cells | 2.45 (1.98 - 3.12) | 3.31 (2.52 - 5.07) | ***<0.05*** | 0.7 |
| CD161^+^, % of CD4 T cells | 12.4 (8.58 - 15.73) | 10.65 (5.8 - 15.2) | 0.66 | 0.96 |
| CXCR3^+^, % of CD4 T cells | 24.72 (19.33 - 32.78) | 19.05 (12.89 - 36.48) | 0.45 | 0.96 |
| Treg, % of CD4 T cells | 5 (4.31 - 5.92) | 4.89 (4.17 - 5.95) | 0.86 | 0.96 |
| CD8^+^, % of T cells | 30.75 (25.1 - 37.02) | 28.95 (24.28 - 32.65) | 0.61 | 0.96 |
| CD27^+^/CD45RA^+^, % of CD8 T cells | 45.15 (38.9 - 56.28) | 49.7 (42.38 - 58.95) | 0.34 | 0.96 |
| CD27^+^/CD45RA^-^, % of CD8 T cells | 27 (14.72 - 34.6) | 23.7 (20.45 - 29.02) | 0.38 | 0.96 |
| CD27^-^/CD45RA^-^, % of CD8 T cells | 9.18 (3.84 - 16.6) | 10.11 (6.58 - 15.22) | 0.88 | 0.96 |
| CD27^-^/CD45RA^+^, % of CD8 T cells | 12.25 (5.44 - 27.88) | 9.7 (3.17 - 21.2) | 0.85 | 0.96 |
| CD28^+^, % of CD8 T cells | 81.65 (69.88 - 85.9) | 81.45 (64.78 - 93.35) | 0.73 | 0.96 |
| CD85^+^, % of CD8 T cells | 11.2 (5.34 - 23.88) | 11.55 (7.33 - 21.8) | 0.43 | 0.96 |
| PD-1^+^, % of CD8 T cells | 15.95 (12.58 - 23.42) | 19.55 (14.3 - 23.1) | 0.94 | 0.96 |
| HLA-DR^+^/CD38^+^, % of CD8 T cells | 6 (4.3 - 8.2) | 9.79 (4.47 - 11.05) | ***0.04*** | 0.7 |
| CD161^+^, % of CD8 T cells | 10.5 (7.25 - 11.72) | 11.6 (8.07 - 16.72) | 0.52 | 0.96 |
| CXCR3, % of CD8 T cells | 38.26 (32.44 - 43.13) | 40.7 (36.04 - 74.52) | 0.42 | 0.96 |

^a^p-values are corrected for BMI; ^b^q-value calculated using false discovery rate

**Table S3. Mann-Whitney rank-based analysis of phosphorylation of signaling transducers after various stimulation of immune cells in vitamin D sufficient and deficient individuals.**

| **Cell type** | **Stimulator** | **Phosphorylation change** | **Vitamin D Severely Deficient Median (IQR)** | **Vitamin D Sufficient Median (IQR)** | **p-value^a^** | **q-value^b^** |
| --- | --- | --- | --- | --- | --- | --- |
| B Cells | IFN-α | STAT1 | 1.27 (1.21 - 1.43) | 1.23 (1.19 - 1.27) | 0.32 | 0.56 |
| - | - | STAT3 | 1.59 (1.33 - 1.71) | 1.26 (1.21 - 1.49) | 0.05 | 0.19 |
| - | - | STAT5 | 1.16 (1.12 - 1.23) | 1.12 (1.09 - 1.19) | 0.35 | 0.57 |
| - | IFN-γ | STAT1 | 1.49 (1.4 - 1.61) | 1.51 (1.46 - 1.54) | 0.67 | 0.82 |
| - | - | STAT3 | 1.12 (1.09 - 1.14) | 1.14 (1.09 - 1.15) | 0.72 | 0.82 |
| - | - | STAT5 | 1.18 (1.16 - 1.2) | 1.2 (1.17 - 1.22) | 0.3 | 0.56 |
| - | IL-2 | STAT1 | 1.4 (1.35 - 1.46) | 1.52 (1.48 - 1.52) | ***<0.01*** | ***0.03*** |
| - | **-** | STAT3 | 1.45 (1.42 - 1.48) | 1.47 (1.44 - 1.5) | 0.77 | 0.84 |
| - | **-** | STAT5 | 1.21 (1.18 - 1.23) | 1.2 (1.17 - 1.24) | 0.7 | 0.82 |
| - | IL-6 | STAT1 | 1.02 (1 - 1.04) | 1.06 (1.03 - 1.07) | 0.02 | 0.16 |
| - | **-** | STAT3 | 1.1 (1.06 - 1.13) | 1.14 (1.1 - 1.15) | 0.22 | 0.48 |
| - | **-** | STAT5 | 1.02 (1 - 1.04) | 1.02 (1.01 - 1.03) | 0.81 | 0.84 |
| - | IL-7 | STAT1 | 1.07 (1.05 - 1.09) | 1.1 (1.09 - 1.12) | 0.05 | 0.19 |
| - | **-** | STAT3 | 1.1 (1.09 - 1.15) | 1.15 (1.11 - 1.16) | 0.2 | 0.48 |
| - | **-** | STAT5 | 1.05 (1.02 - 1.08) | 1.05 (1.04 - 1.06) | 0.84 | 0.84 |
| - | IL-10 | STAT1 | 1.14 (1.09 - 1.15) | 1.2 (1.19 - 1.23) | ***<0.01*** | ***0.02*** |
| - | **-** | STAT3 | 1.98 (1.77 - 2.12) | 1.78 (1.61 - 1.94) | 0.07 | 0.21 |
| - | **-** | STAT5 | 1.02 (1.01 - 1.04) | 1.04 (1.03 - 1.05) | 0.13 | 0.34 |
| - | IL-21 | STAT1 | 1.23 (1.18 - 1.25) | 1.28 (1.24 - 1.3) | 0.03 | 0.19 |
| - | **-** | STAT3 | 1.81 (1.55 - 2.02) | 1.62 (1.53 - 1.71) | 0.31 | 0.56 |
| - | **-** | STAT5 | 1.09 (1.07 - 1.12) | 1.09 (1.05 - 1.1) | 0.59 | 0.79 |
| CD4^+^ T cells | IFN-α | STAT1 | 1.5 (1.4 - 1.77) | 1.58 (1.37 - 1.63) | 0.79 | 0.92 |
| - | - | STAT3 | 1.58 (1.45 - 1.87) | 1.55 (1.25 - 1.63) | 0.28 | 0.48 |
| - | - | STAT5 | 1.4 (1.35 - 1.59) | 1.46 (1.27 - 1.67) | 0.97 | 0.97 |
| - | IFN-γ | STAT1 | 1.32 (1.26 - 1.33) | 1.37 (1.35 - 1.38) | ***<0.01*** | ***0.01*** |
| - | - | STAT3 | 1.12 (1.1 - 1.15) | 1.17 (1.15 - 1.17) | 0.04 | 0.2 |
| - | - | STAT5 | 1.19 (1.17 - 1.2) | 1.23 (1.18 - 1.24) | 0.11 | 0.3 |
| - | IL-2 | STAT1 | 1.51 (1.41 - 1.51) | 1.59 (1.57 - 1.62) | ***<0.01*** | ***0.03*** |
| - | **-** | STAT3 | 1.62 (1.52 - 1.7) | 1.64 (1.56 - 1.71) | 0.83 | 0.92 |
| - | **-** | STAT5 | 2.06 (1.81 - 2.23) | 2.05 (1.9 - 2.39) | 0.63 | 0.92 |
| - | IL-6 | STAT1 | 1.28 (1.13 - 1.34) | 1.28 (1.15 - 1.32) | 0.97 | 0.97 |
| - | **-** | STAT3 | 2.58 (2.01 - 2.7) | 2.08 (1.53 - 2.35) | 0.08 | 0.24 |
| - | **-** | STAT5 | 1.1 (1.06 - 1.12) | 1.09 (1.07 - 1.14) | 0.8 | 0.92 |
| - | IL-7 | STAT1 | 1.15 (1.1 - 1.18) | 1.17 (1.16 - 1.2) | 0.28 | 0.48 |
| - | **-** | STAT3 | 1.23 (1.22 - 1.28) | 1.29 (1.22 - 1.31) | 0.68 | 0.92 |
| - | **-** | STAT5 | 1.6 (1.48 - 1.88) | 1.93 (1.6 - 2.15) | 0.3 | 0.48 |
| - | IL-10 | STAT1 | 1.18 (1.14 - 1.19) | 1.29 (1.23 - 1.31) | ***0.01*** | ***<0.05*** |
| - | **-** | STAT3 | 2.37 (2.02 - 2.76) | 2.19 (1.83 - 2.36) | 0.21 | 0.48 |
| - | **-** | STAT5 | 1.05 (1.04 - 1.06) | 1.08 (1.07 - 1.09) | 0.08 | 0.24 |
| - | IL-21 | STAT1 | 1.31 (1.25 - 1.34) | 1.37 (1.33 - 1.42) | 0.07 | 0.24 |
| - | **-** | STAT3 | 2.47 (2.25 - 2.78) | 2.33 (1.97 - 2.44) | 0.23 | 0.48 |
| - | **-** | STAT5 | 1.21 (1.18 - 1.22) | 1.19 (1.16 - 1.24) | 0.73 | 0.92 |
| CD8+ T cells | IFN-α | STAT1 | 1.55 (1.4 - 1.85) | 1.56 (1.5 - 3.44) | 0.63 | 0.75 |
| - | - | STAT3 | 1.68 (1.41 - 1.86) | 1.55 (1.29 - 1.66) | 0.27 | 0.7 |
| - | - | STAT5 | 1.39 (1.29 - 1.6) | 1.51 (1.33 - 2.62) | 0.57 | 0.75 |
| - | IFN-γ | STAT1 | 1.25 (1.11 - 1.3) | 1.32 (1.15 - 1.34) | 0.12 | 0.64 |
| - | - | STAT3 | 1.12 (1.1 - 1.17) | 1.12 (1.09 - 1.14) | 0.3 | 0.7 |
| - | - | STAT5 | 1.16 (1.07 - 1.17) | 1.16 (1.09 - 1.19) | 0.4 | 0.71 |
| - | IL-2 | STAT1 | 1.41 (1.34 - 1.47) | 1.54 (1.36 - 1.55) | 0.02 | 0.17 |
| - | **-** | STAT3 | 1.56 (1.48 - 1.88) | 1.55 (1.42 - 1.65) | 0.19 | 0.7 |
| - | **-** | STAT5 | 2.27 (1.76 - 3.42) | 2.13 (1.83 - 3.64) | 0.48 | 0.72 |
| - | IL-6 | STAT1 | 1.22 (1.15 - 1.5) | 1.25 (1.16 - 1.54) | 0.64 | 0.75 |
| - | **-** | STAT3 | 2.35 (2.05 - 2.67) | 2.01 (1.64 - 2.52) | 0.25 | 0.7 |
| - | **-** | STAT5 | 1.07 (1.04 - 2.05) | 1.14 (1.03 - 1.43) | 0.82 | 0.82 |
| - | IL-7 | STAT1 | 1.13 (1.1 - 1.23) | 1.13 (1.12 - 1.21) | 0.47 | 0.72 |
| - | **-** | STAT3 | 1.24 (1.18 - 1.38) | 1.25 (1.16 - 1.29) | 0.34 | 0.7 |
| - | **-** | STAT5 | 1.67 (1.34 - 2.78) | 1.9 (1.54 - 4.54) | 0.28 | 0.7 |
| - | IL-10 | STAT1 | 1.2 (1.15 - 1.44) | 1.26 (1.25 - 1.59) | ***0.02*** | 0.17 |
| - | **-** | STAT3 | 2.83 (2.32 - 3.78) | 2.51 (2.29 - 3.04) | 0.58 | 0.75 |
| - | **-** | STAT5 | 1.07 (1.04 - 1.76) | 1.09 (1.07 - 1.35) | 0.37 | 0.7 |
| - | IL-21 | STAT1 | 1.28 (1.26 - 1.36) | 1.34 (1.31 - 1.54) | 0.02 | 0.17 |
| - | **-** | STAT3 | 2.92 (2.35 - 3.39) | 2.45 (2.37 - 2.79) | 0.73 | 0.76 |
| - | **-** | STAT5 | 1.19 (1.14 - 1.75) | 1.2 (1.16 - 1.53) | 0.69 | 0.76 |
| Monocytes | IFN-α | STAT1 | 1.43 (1.35 - 1.65) | 1.46 (1.39 - 1.96) | 0.49 | 0.78 |
| - | - | STAT3 | 1.42 (1.23 - 1.84) | 1.46 (1.21 - 2) | 0.6 | 0.78 |
| - | - | STAT5 | 1.21 (1.13 - 1.29) | 1.24 (1.17 - 1.54) | 0.26 | 0.64 |
| - | IFN-γ | STAT1 | 1.76 (1.7 - 2.18) | 1.86 (1.69 - 2.74) | 0.37 | 0.7 |
| - | - | STAT3 | 1.26 (1.23 - 1.42) | 1.33 (1.2 - 1.36) | 0.97 | 0.97 |
| - | - | STAT5 | 1.44 (1.4 - 1.55) | 1.51 (1.37 - 1.76) | 0.22 | 0.64 |
| - | IL-2 | STAT1 | 1.45 (1.26 - 1.49) | 1.56 (1.17 - 1.59) | 0.27 | 0.64 |
| - | **-** | STAT3 | 1.72 (1.4 - 1.81) | 1.66 (1.29 - 1.76) | 0.4 | 0.7 |
| - | **-** | STAT5 | 1.3 (1.11 - 1.36) | 1.37 (1.03 - 1.39) | 0.77 | 0.87 |
| - | IL-6 | STAT1 | 1.08 (1.06 - 1.37) | 1.1 (1.08 - 1.11) | 0.15 | 0.51 |
| - | **-** | STAT3 | 1.47 (1.3 - 1.7) | 1.42 (1.3 - 1.52) | 0.78 | 0.87 |
| - | **-** | STAT5 | 1.04 (1.02 - 1.13) | 1.05 (1.02 - 1.08) | 0.73 | 0.87 |
| - | IL-7 | STAT1 | 1.12 (1.09 - 1.19) | 1.14 (1.12 - 1.17) | 0.38 | 0.7 |
| - | **-** | STAT3 | 1.24 (1.17 - 1.32) | 1.28 (1.24 - 1.36) | 0.08 | 0.35 |
| - | **-** | STAT5 | 1.13 (1.09 - 1.18) | 1.16 (1.13 - 1.2) | 0.05 | 0.34 |
| - | IL-10 | STAT1 | 1.31 (1.27 - 1.43) | 1.35 (1.34 - 1.63) | 0.04 | 0.34 |
| - | **-** | STAT3 | 3.31 (2.85 - 3.64) | 2.9 (2.45 - 3.25) | 0.53 | 0.78 |
| - | **-** | STAT5 | 1.12 (1.09 - 1.19) | 1.17 (1.13 - 1.23) | 0.05 | 0.34 |
| - | IL-21 | STAT1 | 1.33 (1.31 - 1.37) | 1.37 (1.35 - 1.41) | 0.06 | 0.34 |
| - | **-** | STAT3 | 1.87 (1.73 - 1.96) | 1.86 (1.73 - 1.97) | 0.91 | 0.95 |
| - | **-** | STAT5 | 1.17 (1.13 - 1.19) | 1.15 (1.12 - 1.22) | 0.59 | 0.78 |
| NK Cells | IFN-α | STAT1 | 1.27 (1.14 - 2.35) | 1.42 (1.2 - 1.75) | 0.2 | 0.35 |
| - | - | STAT3 | 1.34 (1.18 - 1.69) | 1.38 (1.09 - 1.42) | 0.83 | 0.83 |
| - | - | STAT5 | 1.12 (1.07 - 1.35) | 1.17 (1.11 - 1.25) | 0.26 | 0.39 |
| - | IFN-γ | STAT1 | 1.05 (1.04 - 1.05) | 1.05 (1 - 1.06) | 0.01 | 0.06 |
| - | - | STAT3 | 1.01 (1 - 1.01) | 1.01 (1 - 1.02) | 0.68 | 0.73 |
| - | - | STAT5 | 1.05 (1.04 - 1.06) | 1.07 (1.06 - 1.08) | 0.29 | 0.41 |
| - | IL-2 | STAT1 | 1.27 (1.17 - 1.31) | 1.1 (1.06 - 1.23) | ***0*** | 0.06 |
| - | **-** | STAT3 | 2.54 (1.6 - 2.83) | 1.3 (1.27 - 1.52) | ***0.02*** | 0.07 |
| - | **-** | STAT5 | 2.8 (2.07 - 4.65) | 2.74 (1.74 - 4.05) | 0.35 | 0.45 |
| - | IL-6 | STAT1 | 1.22 (1.13 - 1.25) | 1.09 (1.08 - 1.25) | 0.06 | 0.16 |
| - | **-** | STAT3 | 1.37 (1.34 - 1.39) | 1.26 (1.25 - 1.35) | 0.25 | 0.39 |
| - | **-** | STAT5 | 1.08 (1.05 - 1.13) | 1.09 (1.08 - 1.11) | 0.16 | 0.34 |
| - | IL-7 | STAT1 | 1.22 (1.13 - 1.25) | 1.12 (1.1 - 1.24) | 0.03 | 0.09 |
| - | **-** | STAT3 | 1.41 (1.32 - 1.44) | 1.24 (1.23 - 1.28) | 0.04 | 0.11 |
| - | **-** | STAT5 | 1.15 (1.11 - 1.38) | 1.16 (1.11 - 1.21) | 0.69 | 0.73 |
| - | IL-10 | STAT1 | 1.36 (1.32 - 1.39) | 1.24 (1.21 - 1.41) | 0.18 | 0.35 |
| - | **-** | STAT3 | 4.01 (3.57 - 5.82) | 3.93 (3.22 - 4.68) | 0.4 | 0.5 |
| - | **-** | STAT5 | 1.16 (1.15 - 1.17) | 1.14 (1.1 - 1.24) | ***0.01*** | 0.06 |
| - | IL-21 | STAT1 | 1.35 (1.33 - 1.46) | 1.25 (1.22 - 1.34) | 0.13 | 0.3 |
| - | **-** | STAT3 | 3.93 (3.15 - 5.63) | 3.56 (2.77 - 4.23) | 0.47 | 0.55 |
| - | **-** | STAT5 | 1.32 (1.24 - 1.5) | 1.17 (1.15 - 1.25) | ***0.01*** | 0.06 |

^a^p-values are corrected for BMI; ^b^q-value calculated using false discovery rate; significant values are in italics and bolded.
